# Acid Reflux Directly Causes Sleep Disturbances in Rat with Chronic Esophagitis

**DOI:** 10.1371/journal.pone.0106969

**Published:** 2014-09-12

**Authors:** Kenichi Nakahara, Yasuhiro Fujiwara, Takuya Tsukahara, Hirokazu Yamagami, Tetsuya Tanigawa, Masatsugu Shiba, Kazunari Tominaga, Toshio Watanabe, Yoshihiro Urade, Tetsuo Arakawa

**Affiliations:** 1 Department of Gastroenterology, Osaka City University Graduate School of Medicine, Osaka, Japan; 2 Department of Molecular Behavioral Biology, Osaka Bioscience Institute, Suita, Japan; 3 Molecular Sleep Biology Laboratory, International Institute for Integrative Sleep Medicine, World Premier International Research Center, Tsukuba University, Tsukuba, Japan; Shiga University of Medical Science, Japan

## Abstract

**Background & Aims:**

Gastroesophageal reflux disease (GERD) is strongly associated with sleep disturbances. Proton pump inhibitor (PPI) therapy improves subjective but not objective sleep parameters in patients with GERD. This study aimed to investigate the association between GERD and sleep, and the effect of PPI on sleep by using a rat model of chronic acid reflux esophagitis.

**Methods:**

Acid reflux esophagitis was induced by ligating the transitional region between the forestomach and the glandular portion and then wrapping the duodenum near the pylorus. Rats underwent surgery for implantation of electrodes for electroencephalogram and electromyogram recordings, and they were transferred to a soundproof recording chamber. Polygraphic recordings were scored by using 10-s epochs for wake, rapid eye movement sleep, and non-rapid eye movement (NREM) sleep. To examine the role of acid reflux, rats were subcutaneously administered a PPI, omeprazole, at a dose of 20 mg/kg once daily.

**Results:**

Rats with reflux esophagitis presented with several erosions, ulcers, and mucosal thickening with basal hyperplasia and marked inflammatory infiltration. The reflux esophagitis group showed a 34.0% increase in wake (232.2±11.4 min and 173.3±7.4 min in the reflux esophagitis and control groups, respectively; p<0.01) accompanied by a reduction in NREM sleep during light period, an increase in sleep fragmentation, and more frequent stage transitions. The use of omeprazole significantly improved sleep disturbances caused by reflux esophagitis, and this effect was not observed when the PPI was withdrawn.

**Conclusions:**

Acid reflux directly causes sleep disturbances in rats with chronic esophagitis.

## Introduction

Gastroesophageal reflux disease (GERD) is caused by the reflux of gastric contents into the esophagus [1], and it is characterized by typical symptoms such as heartburn and acid regurgitation [2]. GERD is currently the most common gastrointestinal disease encountered in gastroenterology practice [3], [4]. Several studies have shown that GERD is strongly associated with sleep disturbances [5]–[13] resulting in work productivity impairment [7], [9], poor health-related quality of life [6], [10], and daytime sleepiness [8]. Therefore, sleep disturbances in patients with GERD is one of the most important clinical problems. Recent studies suggest that the association between GERD and sleep disturbances is bidirectional [10], [14]; GERD causes sleep problems such as difficulty in falling asleep, frequent awakenings because of nighttime heartburn, early morning awakenings, and poor sleep quality [15]. Additionally, sleep deprivation appears to cause worsening of the symptoms of GERD by promoting esophageal mucosal hypersensitivity against gastric acids [16]. Although several factors are attributable for the association between GERD and sleep disturbances, nighttime reflux is the key factor. However, the concepts that a nighttime reflux event precedes the arousal response [17] and that acid reflux is caused by a transient lower esophageal sphincter relaxation (TLESR) triggered by the awakening [18] remain somewhat controversial.

Proton pump inhibitors (PPIs) are the mainstay treatment for GERD [19], and several clinical trials have reported the effects of PPIs on sleep disturbance in patients with GERD [19]–[23]. In a study of 650 patients with GERD, Johnson et al. showed that esomeprazole significantly relieved nighttime heartburn and GERD-related sleep disturbances as well as improved the sleep quality when compared with a placebo [20]. In another study of 305 patients with GERD, Fass et al. reported that dexlansoprazole (modified release, 30 mg) significantly increased the number of nights without heartburn and improved GERD-related sleep disturbances when compared with a placebo [21]. These large randomized placebo-controlled clinical trials demonstrated that PPIs significantly improved subjective sleep parameters in patients with GERD. Conversely, two studies failed to demonstrate significant improvement of objective sleep parameters in patients with GERD treated with PPIs versus a placebo. By using polysomnography, Orr et al found that rabeprazole improved nighttime GERD symptoms and sleep quality in 28 patients with GERD, but it did not alter objective sleep parameters, such as sleep latency, sleep efficiency, arousals per hour, and the proportion of deeper sleep stages [22]. Similarly, another study demonstrated that esomeprazole significantly improved nighttime GERD events in 15 patients, but it did not affect sleep parameters, such as total sleep time, sleep efficiency, and latency of sleep onset [23]. The explanations for the discrepancy between effects of PPIs on subjective and objective sleep parameters are unknown, but the discrepancy might be owing to the small sample size of these polysomnographic studies. Taken together, PPIs promise improvement of subjective sleep parameters in patients with GERD, but clinical evidence that PPIs improve objective sleep parameters has not yet been confirmed.

Whether acid reflux directly causes sleep disturbances is the most important clinical question, and it has not yet been answered. As numerous factors such as stress, lifestyle, and surrounding circumstances affect sleep status in humans, it is hard to examine the direct effect of acid reflux on the sleep of patients with GERD. Therefore, the aim of the present study was to examine the following by using a rat chronic acid reflux esophagitis model: 1) whether gastroesophageal reflux directly affects sleep; 2) if so, the mechanism of how it affects sleep; and 3) the effects of PPIs on objective sleep parameters.

## Methods

### Animals and Induction of Acid Reflux Esophagitis

**Figure 1:**
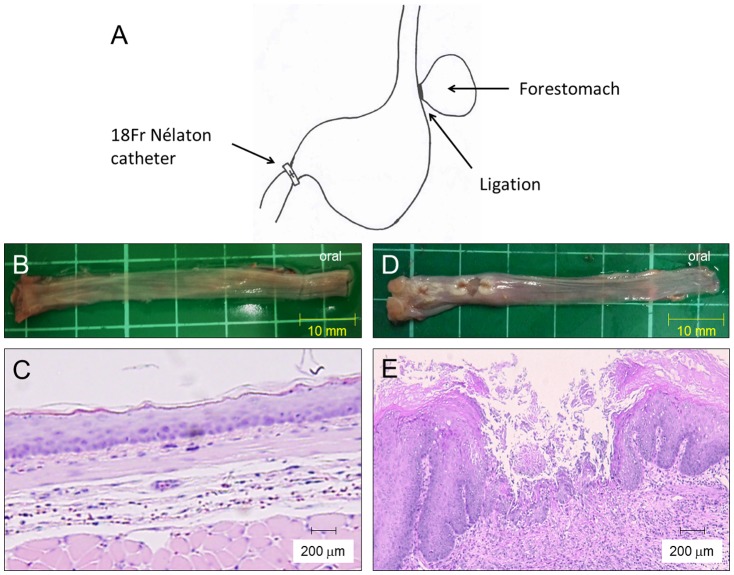
Induction of rat acid reflux esophagitis, macroscopic, and histological findings. (A) A scheme of a rat reflux esophagitis model. (B) Macroscopic appearance of a normal esophagus. (C) Histology of the normal esophagus revealed thin epithelium with few inflammatory cells. (D) Macroscopic appearance of reflux esophagitis showed several erosions and ulcers at the middle and lower esophagus. (E) Mucosal thickening with basal cell hyperplasia and marked inflammatory cell infiltration were observed in reflux esophagitis. (Hematoxylin-eosin staining, original magnification ×200).

The experimental protocol of this study was approved by the Institutional Animal Care and Use Committee of the Osaka City University Graduate School of Medicine. Specific pathogen-free, male Wistar rats (Japan SLC, Hamamatsu, Japan) weighing 250–300 g at the start of the experiment were used. They were housed at a constant temperature (24±0.5°C) with relative humidity (60±2%) in cages with an automatically controlled 12:12-h light/dark cycle (light on at 9:00 a.m.), and they had free access to food and water. The experimental protocol was approved by the Animal Care and Use Committee of the Osaka City University. Acid reflux esophagitis was induced by the method reported by Omura et al [24]. In brief, the duodenum near the pyloric ring was covered with a 2-mm wide piece of an 18-Fr Nélaton catheter (Terumo Co, Tokyo, Japan), and the transitional region between the forestomach and the glandular portion was ligated to enhance reflux of gastric contents into the esophagus (Figure 1A). Sham operated rats were used as controls.

### Electroencephalogram and Electromyogram Recordings

**Figure 2:**
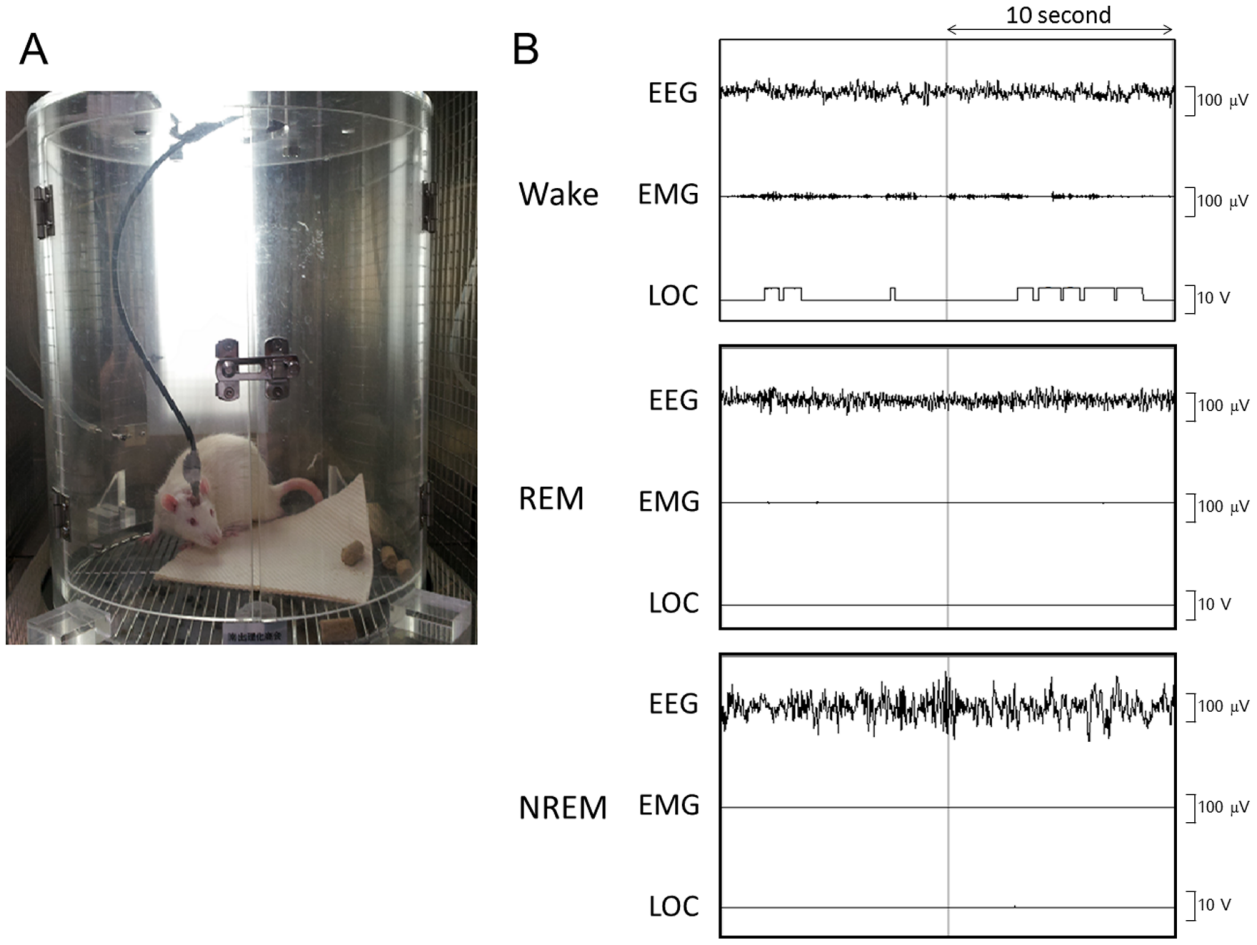
Polygraphic recordings used in this study. (A) Polygraphic system including EEG, EMG, and LOC in a sound-proof recording gage. (B) Typical EEG, EMG, and LOC of wake, REM sleep, and NREM sleep. EEG, electroencephalograph; EMG, electromyography; LOC, locomotor; NREM, nonrapid eye movement; REM, rapid eye movement.

Ten days after induction of reflux esophagitis, rats underwent surgery for implantation of electrodes for electroencephalogram (EEG) and electromyogram (EMG) recordings. The electrodes were fixed on the skull by using dental cement, and 4 stainless steel screws were anchored to the skull. Two stainless steel wire electrodes for EMG recordings were placed into the neck muscles. Postoperatively, each rat was allowed 10 days of recovery, and it was then transferred to a soundproof recording chamber and connected to an EEG/EMG recording cable for 3 days of habituation to experimental conditions (Figure 2A). This chamber included infrared sensors to detect body movement. The EEG/EMG signals were amplified and filtered (EEG, 0.5–30 Hz; EMG, 16–128 Hz), then digitized at a sampling rate of 128 Hz and recorded by using a data acquisition program SLEEPSIGN software (Kissei Comtec, Nagano, Japan) [25], [26]. Experimental recordings were taken for each rat during 3 consecutive 24-h periods, starting at 9:00 a.m. After completion, polygraphic recordings were automatically scored offline by using 10-s epochs for wake, rapid eye movement (REM) sleep, and non-rapid eye movement (NREM) sleep by SLEEPSIGN according to standard criteria [27], [28]. As a final step, defined sleep-wake stages were visually examined and corrected, if necessary. A sleep stage was characterized as follows: NREM sleep was defined as the presence of high-amplitude slow or spindle waves on the EEG and low voltage activity on the EMG; REM sleep, as low-voltage waves and activity on the EEG and EMG, respectively; and wake, as low-voltage waves on the EEG and high-voltage activity on the EMG. Locomotor (LOC) findings were used as supportive data to confirm each stage. Figure 2B shows the typical EEG/EMG findings of wake, REM sleep, and NREM sleep.

### Assessment of Reflux Esophagitis

After completion of the experiments, rats were killed and the esophagus was gently rinsed with saline. Macroscopic esophageal lesions were confirmed, and the resected tissue was fixed in 10% neutral buffered formalin. Samples were embedded in paraffin, and 4-µm thick sections were prepared. Hematoxylin and eosin staining was performed for standard morphological analysis.

### Effect of PPI on Sleep in Rats with Reflux Esophagitis

Eight rats with reflux esophagitis were used. Ten days after the operation, a vehicle (0.5% of carboxymethyl cellulose) was administered subcutaneously once daily for 2 days at 5:00 p.m. After the administration on the second day, EEG and EMG recordings were noted on the subsequent day (pre-PPI group). Two days after the EEG/EMG recording, rats were subcutaneously administered omeprazole (20 mg/kg) once daily at 5:00 p.m. for 2 days. After the administration on the second day, EEG and EMG recordings were noted on the subsequent day (on-PPI group). In order to examine the effect of PPI withdrawal, EEG and EMG recordings were noted again 3 days after the last administration of omeprazole (post-PPI group).

### Analysis of Gastric Acid Secretion

Gastric acid secretion was measured in rats treated with omeprazole. Omeprazole was subcutaneously administered at a dose of 20 mg/kg at 5:00 p.m. for 2 days, and then, the rats were divided into two groups. In one group, the pylorus was ligated at 9:00 a.m. the next day, and the rats were killed 6 h after ligation (n = 5). In another group, the pylorus was ligated at 3:00 p.m. the next day, and the rats were killed 6 h after ligation (n = 5). The volume of gastric acid secreted was measured, and the concentration of acid was determined by titration with 0.1 N NaOH by using an automatic titration system (AUT-301L, TOA Electronics, Ltd, Tokyo, Japan). The rats administered the vehicle (0.5% carboxymethyl cellulose) were used as controls.

### Statistical Analysis

The data are presented as the mean ± standard error of mean. The statistical significance of the duration of each stage, time course data of sleep-wake profiles, the number of each stage bout, mean duration of each stage, and stage transition were assessed by using the two-tailed unpaired *t*-test or one-way ANOVA followed by the Dunnett post hoc test. The effects of omeprazole on sleep were analyzed by using one-way repeated measures of ANOVA followed by the Dunnett post hoc test. The level of significance was set at a p-value of 0.05 or less.

## Results

### Macroscopic Appearances and Histological Changes in the Rat Esophagus Owing to Acid Reflux

Figure 1B shows the macroscopic appearance of the normal esophagus. The normal esophagus had a thin esophageal epithelium with few inflammatory cells in the submucosa (Figure 1C). In rats with reflux esophagitis (Figure 1D), several erosions and ulcers in the lower and middle part of the esophagus were observed. Histological examination (Figure 1E) revealed mucosal thickening with basal cell hyperplasia and marked infiltration of inflammatory cells to the lamina propria and submucosa. These histological findings were similar to those found in patients with reflux esophagitis. We confirmed these macroscopic and histological changes in all the rats with reflux esophagitis.

### Effects of Acid Reflux Esophagitis on Sleep

**Figure 3:**
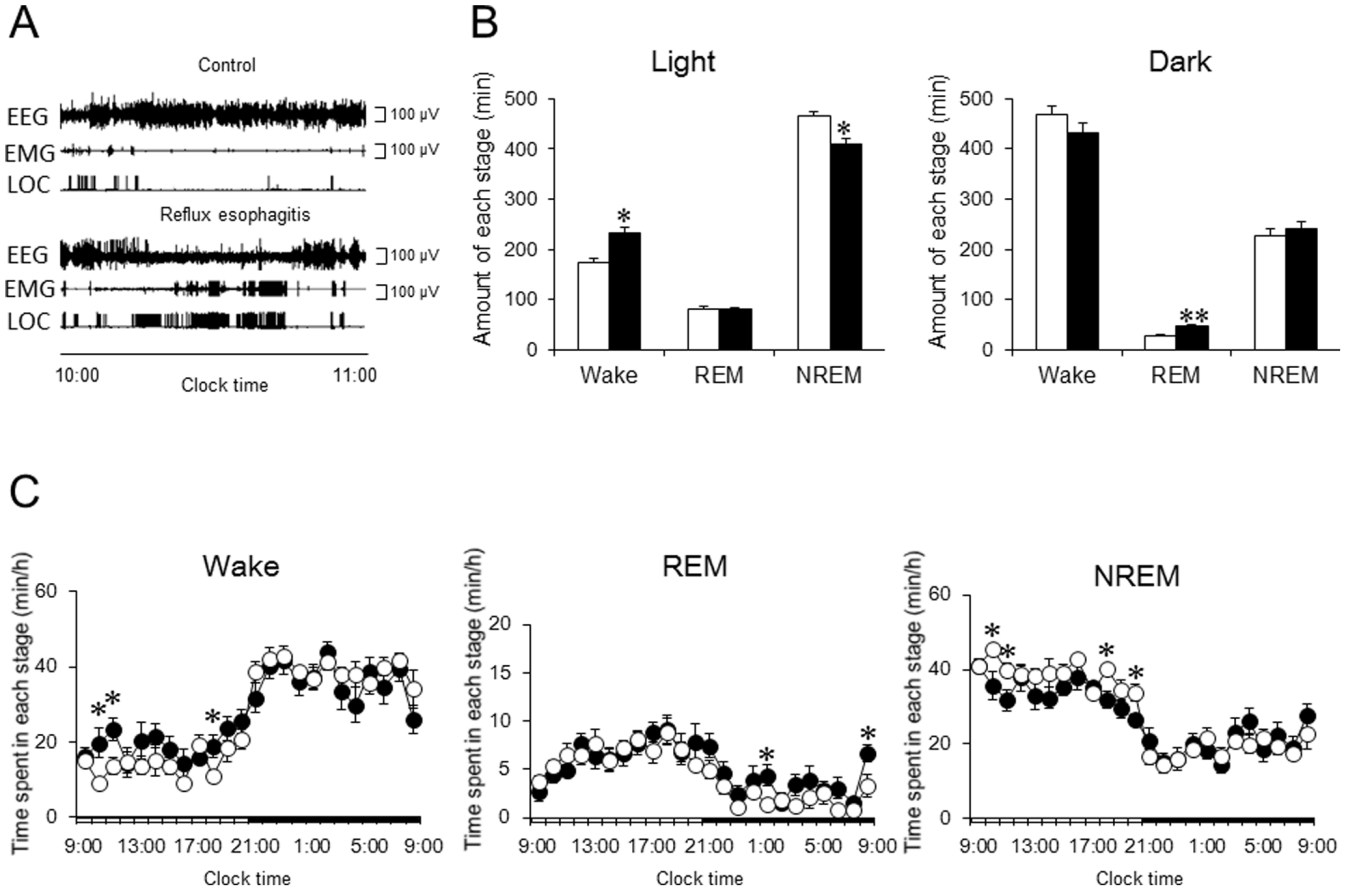
Effect of reflux esophagitis on sleep. (A) Typical EEG, EMG, and LOC of control and reflux esophagitis. (B) Effect of reflux esophagitis on the amount of each stage during the 12-h light period and 12-h dark period. (C) Time course analysis of each stage during a whole day. N = 8. Data are mean ± SEM. White bars and circles represented control rats. Black bars and circles represented reflux esophagitis rats. *p<0.05 versus control. **p<0.01 versus control. EEG, electroencephalograph; EMG, electromyography; LOC, locomotor; NREM, nonrapid eye movement; REM, rapid eye movement.

Figure 3A shows typical polygraphic recordings taken between 10:00 a.m. and 11:00 a.m. for the control rats and rats with reflux esophagitis. Long-term high-amplitude EEG recordings accompanied by low-voltage EMG recordings and a few changes in the LOC signal were found in the control group, while there were several low-amplitude waves on the EEG accompanied by high-voltage EMG recordings and frequent changes in the LOC signal in rats with reflux esophagitis. The total duration of each sleep stage during the light and dark periods is shown in Figure 3B. The reflux esophagitis group presented a 34.0% increase in wake (232.2±11.4 min versus 173.3±7.4 min in the control group, p<0.01) during the light period, accompanied by a reduction in NREM sleep (408.6±12.7 min versus 467.0±6.7 min in the control group, p<0.01), but there was no difference in the total duration of REM sleep between the two groups during the light period (Figure 3B). Such a significant increase in the duration of wake and a significant decrease in the duration of NREM sleep in rats with reflux esophagitis were not observed during the dark period, but the amount of REM sleep significantly increased in rats with reflux esophagitis (Figure 3B). We calculated the time spent in each stage considering 1-h blocks and presented data for the entire day (Figure 3C). Significant differences in wake and NREM sleep between the control and reflux esophagitis groups were found during the early (between 10:00 a.m. and 12:00 p.m.) and late periods (between 6:00 p.m. and 7:00 p.m.) of the light period. REM sleep in rats with reflux esophagitis significantly increased between 1:00 a.m. and 2:00 a.m. and between 8:00 a.m. and 9:00 a.m. (Figure 3C).

### Sleep Fragmentation and Stage Transition in the Rats with Acid Reflux Esophagitis

**Figure 4:**
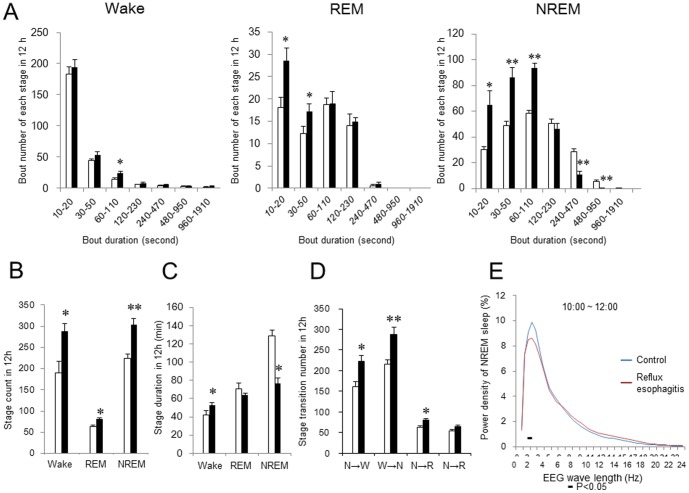
Effect of reflux esophagitis on sleep fragmentation and stage transitions. (A) Number of stage bouts in the 12-h light period. (B) Number of stage counts in the 12-h light period. (C) Duration of each sleep stage in the 12-h light period. (D) Stage transitions during the 12-h light period. NREM, non-rapid eye movement; REM, rapid eye movement. (E) Relative average EEG power density of NREM sleep between 10:00 a.m. and 12:00 a.m. The horizontal bars indicate statistical difference (p<0.05) between control and reflux esophagitis group. N = 8. Data are mean ± SEM. White bars represented control rats and black bars represented reflux esophagitis rats. *p<0.05 versus control. **p<0.01 versus control.

In order to examine sleep fragmentation and stage transition, we further calculated the number of wake, REM sleep, and NREM sleep bouts, and number of stage transitions from NREM to wake, from wake to NREM, from NREM to REM, and from REM to wake. Figure 4 shows the analysis of sleep fragmentation and stage transitions during the light period. The number of short NREM sleep bouts (10–20 s, 30–50 s, and 60–110 s) and REM sleep bouts (10–20 s and 30–50 s) increased in rats with reflux esophagitis compared with controls, while the number of long NREM sleep bouts (240–470 s and 480–950 s) significantly decreased in rats with reflux esophagitis (Figure 4A). The number of wake, REM sleep, and NREM sleep stage counts significantly increased in rats with reflux esophagitis (Figure 4B). The reflux esophagitis group showed a significantly shorter mean duration of NREM sleep (76.9±5.7 s versus 128.9±6.0 s in the control, p<0.01) and a longer mean duration of wake (52.8±2.5 s versus 41.9±5.1 s in the control, p<0.05), but there was no difference in the mean duration of REM sleep between the two groups (Figure 4C). The reflux esophagitis group presented a disruption of the sleep pattern, resulting in significantly more frequent stage transitions from NREM sleep to wake, from wake to NREM sleep, and from NREM to REM sleep (Figure 4D). We analyzed sleep fragmentation and stage transitions during the dark period, but there were no significant differences between the control and reflux esophagitis groups (data not shown). We further analyzed the EEG power spectra during NREM sleep in the early light period in control rats and rats with reflux esophagitis. The power of each 0.5-Hz bin was first averaged across the sleep stages individually and then normalized as a group by calculating the percentage of each bin from the total power (0–24.5 Hz) of each animal. The power density on EEG significantly increased in the frequency range of 2 Hz in the control rats when compared with the rats with reflux esophagitis.

### Effect of PPI on the Volume of Gastric Juice and Acidity

Table 1 shows the effect of omeprazole on the gastric volume, pH, and total acidity. The rats treated with omeprazole showed significant decreases in gastric volume and acid output, and an increase in pH values when compared with the controls. Significant acid suppression was observed between both 9:00 a.m. and 3:00 p.m. and between 3:00 p.m. and 9:00 p.m., suggesting that the dose of omeprazole used in the present study was sufficient to suppress gastric acid secretion during the sleep study period.

**Table 1:** Effects of omeprazole on gastric acid secretion in rats.

	9:00 a.m.–3:00 p.m.			3:00 p.m.–9:00 pm		
	Volume (mL/6 h)	pH	Acid output (µEq/6 h)	Volume (mL/6 h)	pH	Acid output (µEq/6 h)
Control	17.4±1.5	1.4±0.02	1194±124	10.8±2.5	1.5±0.1	897±310
Omeprazole	8.5±0.6*	6.0±0.1*	26±17*	4.4±1.3*	5.9±0.9*	40±34*

Data were mean ± SEM. * p<0.01 versus control.

### Effect of PPI on Sleep in Rats with Acid Reflux Esophagitis

**Figure 5:**
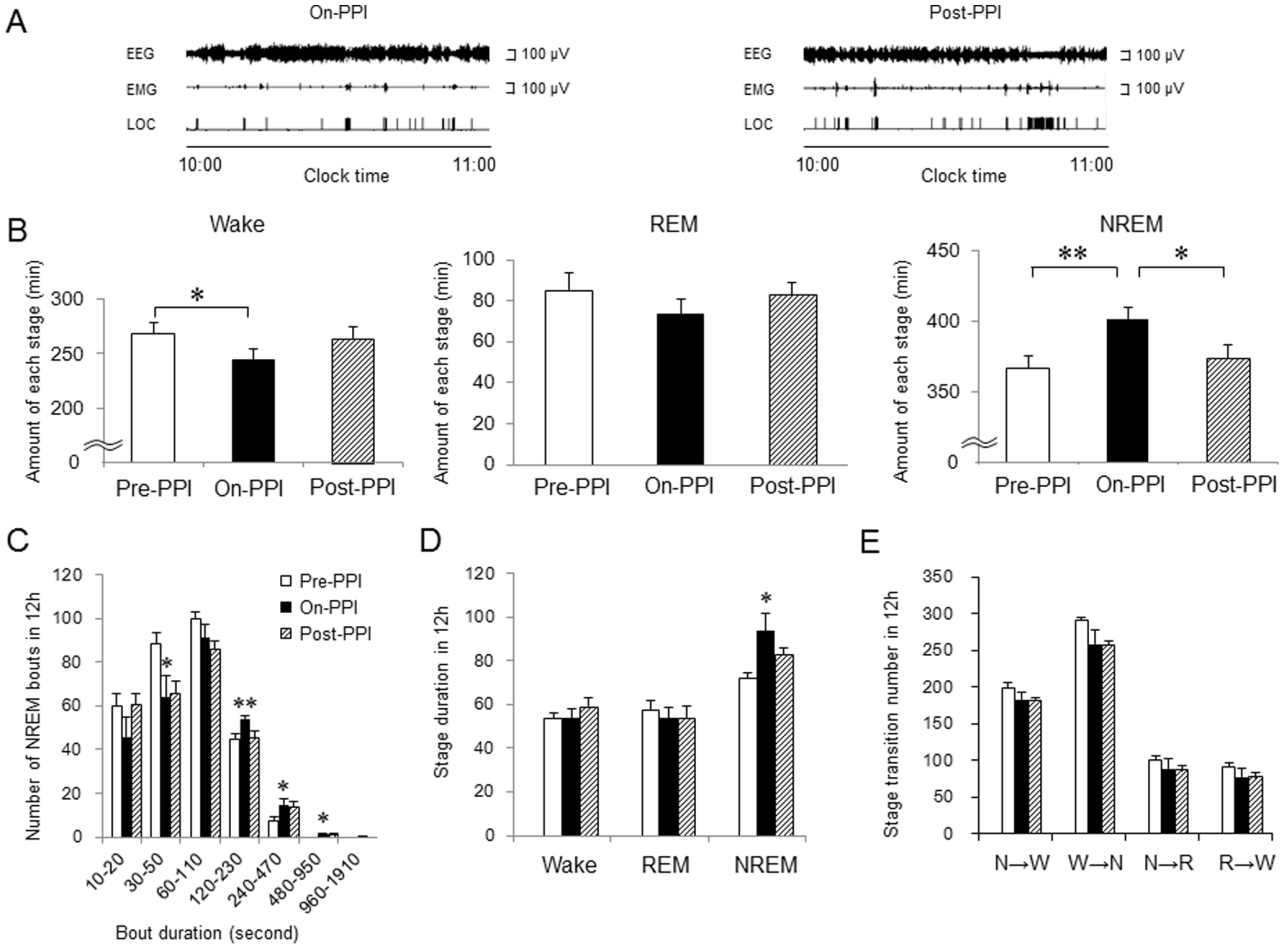
Effect of proton pump inhibitor on sleep disturbances in rats with reflux esophagitis. (A) Typical polygraphic recordings of the on-PPI group and the post-PPI group. (B) Amount of each stage for the 3 groups (pre-PPI group, on-PPI group, and post-PPI group) in the 12-h light period. (C) Number of NREM sleep bouts for the 3 groups in the 12-h light period. (D) Duration of each stage for the 3 groups in the 12-h light period. (E) Stage transitions for the 3 groups during the 12-h light period. N = 7. Data are mean ± SEM. *p<0.05 versus before PPI group. **p<0.01 versus before PPI group.

Figure 5A shows typical data of the EEG and EMG recordings and the LOC findings in rats with reflux esophagitis of the on-PPI and post-PPI group. High-amplitude waves on the EEG and low-voltage activity on the EMG were more frequently observed in the on-PPI group compared with the pre-PPI group and the post-PPI group. The effect of omeprazole on the number of stages, number of NREM bouts, duration of each stage, and stage transition during the light period are shown in Figure 5B-E. A 9.6% increase in NREM sleep was observed after the administration of omeprazole (361.7±10.4 min in the pre-PPI group versus 402.1±8.1 min in the on-PPI group, p<0.01) accompanied by a reduction in wake stage (267.8±11.0 min in the pre-PPI group versus 244.3±10.4 min in the on-PPI group, p<0.05) (Figure 5B). The post-PPI group presented a decrease of 10.1% in NREM sleep compared with the on-PPI group (368.8±10.8 min in the post-PPI group, p<0.05), suggesting that the sleep improvement effects resulting from omeprazole were abolished after withdrawal of the PPI. Although the amount of wake in the post-PPI group tended to be longer than that in the on-PPI group, it did not reach statistical significance (p>0.05). The on-PPI group presented with an increased number of long NREM sleep bouts, ranging 120–230 s, 240–470 s, and 480–950 s, but a decreased number of short NREM sleep bouts, ranging 30–50 s, compared with the pre-PPI group (Figure 5C). Such significant changes in NREM sleep bouts were not observed in the post-PPI group (Figure 5C). There were no differences of REM sleep bouts and wake bouts between the three groups (data not shown). The mean duration of NREM sleep in the on-PPI group was longer than in the pre-PPI group (72.5±2.1 s in the pre-PPI group versus 91.9±6.8 s in on-PPI group, p<0.05), but such an increase in NREM duration was not observed in the post-PPI group. There were no differences in the mean durations of REM sleep and wake stage (Figure 5D) and number of stage transitions (Figure 5E) between the three groups. Such significant differences in the number of stages, number of NREM sleep bouts, and duration of NREM sleep between the three groups were not observed during the dark period (data not shown).

## Discussion

To the best of our knowledge, this is the first report to confirm the direct link between GERD and sleep disturbances. Our study clearly demonstrated that reflux esophagitis induced a significant reduction in the duration of NREM sleep accompanied by an increase in the duration of wake stages during light period in a rat model (Figure 3).

A clinical study involving the use of pH monitoring and actigraphy showed that conscious awakenings associated with reflux events during sleep are seldom symptomatic [29], suggesting that arousals during sleep because of nighttime heartburn could not adequately explain the association between GERD and sleep disturbances. There were two patterns of nighttime reflux in the absence of GERD symptoms, as follows: a typical long reflux event without awakening and a very short amnestic reflux event with consciousness awakening. The latter reflux pattern was associated with poorer sleep quality because of frequent amnestic arousals, leading to sleep fragmentation [30]. Similarly, we found that reflux esophagitis led to a significant increase in short NREM sleep and REM sleep bouts, significant decrease in long NREM sleep bouts (Figure 4A), and more frequent stage transitions from NREM sleep to wakefulness and from NREM to REM sleep (Figure 4D). These findings indicated the presence of sleep fragmentation and poor sleep quality in this rat model of reflux esophagitis.

Allen et al. [31] demonstrated that increased acid reflux in the early recumbent period occurs primarily during the recumbent-awake and not during the recumbent-asleep periods, suggesting that nighttime reflux is associated with difficulty in falling asleep. In a study on patients with GERD, Poh et al. [32] found an increase in the frequency of reflux events in the early morning (immediately after awakening from sleep) in approximately half of the patients with GERD. Interestingly, although rats have quite a different sleep pattern compared to humans, wakefulness in rats with reflux esophagitis increased in the early and late phases of the light period, which corresponds to nighttime in humans (Figure 3C). Further studies would be necessary to evaluate the association between reflux esophagitis and the circadian rhythm. The significant increase in the duration of REM sleep in rats with reflux esophagitis during the dark period, compared with controls (Figure 3C), might be attributable to a compensation for the insufficient amount of NREM sleep during the light period. Such a phenomenon is known as daytime sleepiness in humans.

After the administration of omeprazole at a dose that completely suppressed gastric acid secretion (Table 1), a significant increase in NREM sleep with reduction of the wake stage as well as reduced sleep fragmentation caused by reflux esophagitis was observed (Figure 5B, 5C). Upon the withdrawal of omeprazole treatment, the objective sleep parameters returned to the basal level observed in rats before treatment; therefore, acid reflux was directly associated with sleep disturbances. The reasons why omeprazole did not alter stage transitions are unknown, but this might be attributable to the duration of the PPI treatment. Although short-term PPI administration was used to examine the role of acid reflux in direct association with GERD and sleep disturbances in this study, long-term PPI administration might improve sleep stage transitions caused by acid reflux esophagitis.

The mechanisms of acid reflux that lead to sleep disturbances are discussed below. It is known that TRPV1-immunoreactive sensory nerve fibers are expressed in human esophageal mucosa, and their expression increases in reflux esophagitis [33]. Acid-induced activation of TRPV1 causes ATP release from esophageal epithelial cells, and it induces the release of calcitonin gene-related peptide and substance P from esophageal submucosal neurons [34]. Oshima et al. [35] examined the role of NK-1 receptors (to which substance P binds) in the voluntary movements of rats with chronic reflux esophagitis by using the same model used in the present study. They found that NK-1 receptor antagonists significantly suppressed the reduction of movement caused by reflux esophagitis, suggesting that NK-1 receptors and their related peptides play a role in voluntary movement of rats. The expression of acid-sensing receptors and related peptides in the esophagus has been well studied, as well as their signals related to the central nervous system for perception of heartburn; however, precise interaction between these pathways and sleep are unknown.

Several studies have reported the associations between sleep disturbances and neuropathic pain in animal models [36], [37]. For example, sciatic nerve-ligated animals presented with a significant increase in blood oxygenation level-dependent signal intensity in the pain matrix on functional magnetic resonance imaging scans as well as a statistically significant increase in wakefulness and a decrease in NREM sleep during the light period on EEG/EMG [37]. In such models, neuropathic pain induced an increase in glutamine levels in the cingulate cortex and morphologic changes in astrocytes [38], [39]. Then, the membrane-bound γ-aminobutyric acid (GABA) transporter-3 was exposed on activated astrocytes and suppressed GABAergic neurons; this resulted in sleep disturbances [40]. Future studies are required to determine the association of GABA levels in the cingulate cortex of rats with reflux esophagitis.

In healthy adults, TLESR is the main cause of nighttime reflux [41], but other mechanisms such as strain reflux and free reflux are involved in nighttime reflux of patients with GERD [42]. The rat model for reflux esophagitis used in this study was not related to TLESR, but it was related to mechanical reflux such as strain reflux. Therefore, we could not assess the role of TLESR in sleep disturbances. However, our methods were sufficient to achieve the study purpose, i.e., we demonstrated that reflux events, especially acid reflux, are directly associated with sleep disturbances. We used a specific EEG/EMG recording system to evaluate sleep stages in rats. This system is highly accepted for sleep experiments in several animal models [25]–[28]; therefore, we considered it the best tool to analyze the association between GERD and sleep disturbances.

In conclusion, reflux esophagitis caused sleep disturbances, including an increase in the duration of wake stages, a decrease in the duration of NREM sleep, an increase in sleep fragmentation, and frequent stage transitions. As the PPI omeprazole improved sleep disturbances associated with reflux esophagitis, we concluded that acid reflux directly causes sleep disturbances.
